# Young People’s, Parents’, and Professionals’ Views on Required Components of Mobile Apps to Support Self-Management of Juvenile Arthritis: Qualitative Study

**DOI:** 10.2196/mhealth.9179

**Published:** 2018-01-19

**Authors:** Jennifer M Waite-Jones, Rabiya Majeed-Ariss, Joanna Smith, Simon R Stones, Vanessa Van Rooyen, Veronica Swallow

**Affiliations:** ^1^ School of Healthcare University of Leeds Leeds United Kingdom; ^2^ School of Psychological Sciences University of Manchester Manchester United Kingdom; ^3^ Leeds Children’s Hospital Leeds United Kingdom; ^4^ Faculty of Science Charles Sturt University Bathurst Australia

**Keywords:** Adolescent, young people, juvenile arthritis, mobile apps, self-management, qualitative research

## Abstract

**Background:**

There is growing evidence that supporting self-management of Juvenile Arthritis can benefit both patients and professionals. Young people with Juvenile Arthritis and their healthy peers increasingly use mobile technologies to access information and support in day-to-day life. Therefore, a user-led, rigorously developed and evaluated mobile app could be valuable for facilitating young people’s self-management of Juvenile Arthritis.

**Objective:**

The objective of this study was to seek the views of young people with Juvenile Arthritis, their parents or carers, and health care professionals (HCPs) as to what should be included in a mobile app to facilitate young people’s self-management of chronic Juvenile Arthritis.

**Methods:**

A qualitative approach was adopted with a purposeful sample of 9 young people aged 10-18 years with Juvenile Arthritis, 8 parents or carers, and 8 HCPs involved in their care. Data were gathered through semi-structured focus group and individual interviews with young people and their parents or carers and HCPs. Interview discussion was facilitated through demonstration of four existing health apps to explore participants’ views on strengths and limitations of these, barriers and facilitators to mobile app use, preferred designs, functionality, levels of interaction, and data sharing arrangements. Data were analyzed using the framework approach.

**Results:**

Analysis revealed three interlinked, overarching themes: (1) purpose, (2) components and content, and (3) social support. Despite some differences in emphasis on essential content, general agreement was found between young people with Juvenile Arthritis their parents or carers, and professionals that a mobile app to aid self-management would be useful. Underpinning the themes was a prerequisite that young people are enabled to feel a sense of ownership and control of the app, and that it be an interactive, engaging resource that offers developmentally appropriate information and reminders, as well as enabling them to monitor their symptoms and access social support.

**Conclusions:**

Findings justify and pave the way for a future feasibility study into the production and preliminary testing of such an app. This would consider issues such as compatibility with existing technologies, costs, age, and cross-gender appeal as well as resource implications.

## Introduction

The self-management support needs of young people with long-term conditions (LTCs) such as juvenile arthritis are now better understood [1]. They include the following core skills: problem solving, decision making, resource utilization, and patient and professional relationship formation [2]. Learning the skills and knowledge needed to manage treatment regimens effectively is a key component of LTC management for young people with Juvenile Arthritis as they grow up and transition to adult health care [3-6]. Acquisition of such skills is particularly important during this period of transition, given the changes within previous support structures which included parents or carers and pediatric focused health care professionals (HCPs). Foster et al [7] propose that professionals should encourage young people to take a lead role in managing their condition while supporting parents or carers in the changes to their previous roles and responsibilities.

Stinson et al [8] describe self-management as taking responsibility for symptoms and treatment of a chronic condition, including the ability to cope with the psychological and physical changes and the effects of adopting a more appropriate lifestyle. Coping methods involved gaining information, learning appropriate behavior changes, and improving self-efficacy.

Improved self-efficacy is essential to the ability to be able to self-manage a chronic condition. The concept of self-efficacy can be understood in terms of social cognitive theory developed by Bandura [9,10], which suggests that to successfully promote positive behavior change, there is a need to encourage individuals to gain mastery over their situation through verbal persuasion, improved communication, and vicarious learning from role models. To improve self-efficacy, according to Schwartz et al [3], reciprocal relationships need to be encouraged between patients, parents or carers, and professionals to foster good communication and the motivation for patients to achieve appropriate goals and improve knowledge. Elwyn et al [11] suggest that through sharing decision making within reciprocal relationships, supporting patients, and helping them make decisions based on informed preferences promotes successful self-management.

Furthermore, facilitation of such learning must be carefully tailored to young people’s needs, reflecting a growing evidence base, which suggests that brain development continues up until a young person is in their early twenties [12]. Therefore, young people need support and guidance to become accountable for their own health, enabling them to develop into independent, empowered, and responsible adults [7].

The Internet is becoming the preferred method for young people when seeking health information [13], particularly when searching for tools enabling self-management of chronic conditions [13]. Web-based self-management strategies have been reported by young people to be *a promising avenue* to support them in managing their symptoms and control their emotions as well as access information and social support [8]. Mobile apps can also provide information and integrate health care tasks [14]. Therefore, self-management tools provided by mobile apps could help meet the requirements of young people with Juvenile Arthritis and those involved in their care [15].

Such apps need to be engaging and have usability as well as functionality [16]. The use of mobile apps to improve pain management in young people with Juvenile Arthritis has been studied [17], but there is a lack of rigorously developed apps to support other aspects of self-management, and this suggests a need for further evaluation through rigorous research to ensure best practice [18].

This study completes stage three of the modeling stage [19] of development and evaluation of a mobile app for self-management by young people with Juvenile Arthritis. The first stage involved a user-led Web-based survey of 14 young people with juvenile arthritis (12 females and 2 males aged between 11 and 22 years) to assess the need for a self-management mobile app. A total of 12 respondents felt there was a need for the mobile app and 2 were unsure. Components specified included the following: appointment and treatment reminders, a tool to record/monitor disease activity and quality of life, a goal-setting facility, and a secure central point to share experiences with peers. Places where respondents said they would use such an app included the following: home, school, shopping, hospital, and when with friends [20].

Stage two included a systematic review of the effectiveness of apps for self-management by young people with physical LTCs. Few studies were found to use a systematic, young-person and family-led approach to app development and evaluation, thus confirming the need for this study [14].

The third stage included a qualitative study to understand the preferred components of a mobile app for young-people with juvenile arthritis, their parents or carers, and professionals before developing an app.

The objective of this study was to seek the views of young people with Juvenile Arthritis, their parents or carers, and HCPs as to what should be included in a mobile app to facilitate young people’s self-management of chronic rheumatic disease.

## Methods

### Design

This study was informed by previous stages of the project as well as an additional electronic scoping exercise guided by the Cochrane Handbook [21]. A qualitative design was adopted, which investigated a potential aid to self-management of young people’s Juvenile Arthritis based on the concept of self-efficacy [9,10], and the Medical Research Council [19] framework for the development and evaluation of complex interventions was followed.

### Ethics

Ethical approval was obtained from the National Health Service Health Research Authority (reference no: 193786).

### Participants

Purposeful sampling of young people aged 10-18 years from the database of a pediatric rheumatology clinic based in a large teaching hospital in the north of England catering for different forms of Juvenile Arthritis was carried out by a rheumatology nurse specialist (VVR). To be eligible for recruitment, participants sought had to have been diagnosed with a chronic rheumatic disease. Although the composition of the group depended upon those who agreed to participate, an attempt was made to achieve variation regarding age, developmental stage, disease type and duration, ethnicity, sex, socioeconomic status, and treatment type.

VVR also invited parents or carers and professionals caring for these young people to participate. The final sample group comprised 25 participants, including 9 young people, 8 parents or carers, and 8 HCPs (Table 1). The HCP group comprised consultants, clinical nurse specialists, a psychologist, a youth worker, and a pharmacist.

Verbal and written information (in the form of approved developmentally appropriate information sheets, topic guides, and consent or assent forms) was offered to potential participants. Participants aged 16 years and older provided VS and JW-J with written consent. Patients under 16 years signed assent forms, and their parents or carers signed consent forms on their behalf [22].

**Table 1:** Participants' demographics. HCP: health care professional; PC: parents or carers; YP: young people.

Participant and participant number		Sex	Age (years)
**Young people (N=9)**			
	YP1	Female	17
	YP2	Male	14
	YP3	Female	13
	YP4	Female	11
	YP5	Female	14
	YP6	Female	13
	YP7	Female	10
	YP8	Male	15
	YP9	Female	15
**Parents or carers (N=8)**			
	PC1	Female	N/A^a^
	PC2	Female	N/A
	PC3	Male	N/A
	PC4	Female	N/A
	PC5	Male	N/A
	PC6	Female	N/A
	PC7	Female	N/A
	PC8	Female	N/A
**Health care professionals (N=8)**			
	HCP1	N/A	N/A
	HCP2	N/A	N/A
	HCP3	N/A	N/A
	HCP4	N/A	N/A
	HCP5	N/A	N/A
	HCP6	N/A	N/A
	HCP7	N/A	N/A
	HCP8	N/A	N/A

^a^N/A: not applicable.

### Tools

Developmentally appropriate topic guides (see Multimedia Appendix 1) were used to structure focus groups and individual interviews [23]. Four existing self-management apps were also demonstrated. Two of these were designed for adults with rheumatoid arthritis [24,25], the third was for adults with chronic pain [26], and the fourth was aimed at younger people with type 2 diabetes mellitus [27]. These particular apps were selected on the advice of the user ambassador and coapplicant (SS) (in line with our philosophy of an evidence-based user-led approach, as recommended within our recent systematic review [14]) who thought they offered specific systems relevant to Juvenile Arthritis.

### Data Collection

Using a participatory approach [28], for participants’ convenience, semi-structured interviews were conducted with young people and their parents or carers, and information gathered from the professionals via 2 focus groups. Although offered the opportunity to be interviewed separately from their parent or carer, only one young person expressed a desire for an independent interview and this was only because they were available at a different time to their parents. Developmentally appropriate topic guides were used to explore participants’ information needs, experience of using mobile apps, and opinions of relevant current mobile apps. The sample apps were demonstrated to generate discussion around participants’ views on strengths and limitations of existing mobile apps, barriers and facilitators to mobile app use, preferred designs, functionality, levels of interaction, and data sharing arrangements. Participants were then asked for additional comments. On completion of interviews, the young participants were sent a £10 thank you voucher, and the downloaded information from support groups was posted to young people and parents or carers. Focus groups and interviews lasted between 35 and 60 min and were digitally recorded and transcribed by the first author.

### Data Analysis

Data were analyzed by 5 members of the research team and included 2 children’s nurse researchers and 2 psychologists specializing in children’s health who all have considerable experience in different forms of analysis used in qualitative studies. In addition, the user ambassador and fellow researcher was also involved in each stage of the research project, including analysis. The framework approach was used as an analytic method as this flexible, systematic, and rigorous method offers clarity, transparency, and an audit trail [29-31]. Two transcripts were initially thematically coded by 5 members of the research team, and 4 overarching themes with related subthemes emerged. These provided a framework through which all the transcripts were analyzed and displayed via a bespoke matrix in Microsoft Excel [32]. Once all transcripts had been analyzed, themes were discussed, refined, and critically evaluated resulting in 2 of the original themes being integrated. All final themes, subthemes, and relevant quotations were then reviewed by the whole team and a consensus was achieved that they clearly reflected participants’ views.

## Results

Findings were grouped into 3 interlinked, overarching themes: (1) purpose, (2) components and content, and (3) social support (Textbox 1).

**Core themes and subthemes.**

Purpose

- App ownership
- Monitoring chronic rheumatic disease (Juvenile Arthritis) and information sharing
- Facility for reminders

Components and content

- Desired components
- Essential content
- Practical considerations

Social support (emotional and practical)

- Access and signposting to existing support networks
- Secure peer support
- Understanding from others without Juvenile Arthritis
- Parent support network

### Theme 1: Purpose of Mobile Apps

#### App Ownership

Young people stressed the importance of app ownership and being able to choose if and how they would use it:

Something that you don’t have to get from your doctor...You can use it or not use it...not be forced to.

YP1, young person #1

Overall, a personalized approach was preferred so that individuals could feel supported and empowered by the app:

Yes, it is like there is action. It is like “Here we are here for you!”

YP5

Coded access was suggested by young people and parents or carers to ensure the app provided a secure, supportive environment:

So that people who do not have the code and do not have arthritis can’t get on, so it is as easy as that.

YP1

Although professionals agreed that some form of secure access was needed, they also commented on *good* uses of the app and expressed some concern relating to young people’s desires for ownership:

We would have to be careful because it only takes someone with an app joining a support group and they can easily access cards, you know things that they can use and have an understanding of the system to manufacture a diagnosis of arthritis...We have come across a lot of kids who have said that they have arthritis that it is clear that they don’t.

HCP2

#### Monitoring Juvenile arthritis and Information Sharing

Most participants thought that monitoring Juvenile Arthritis and pain levels would be useful for young people, as illustrated by the following response to the demonstration of an existing app:

That tracking your pain thing [is useful] so that you can monitor [pain].

YP7

And the suggestion that:

You could have a little diary in there as well so if you want to write about your day, because if it helps you and you prefer to write, then maybe you could just type it in and it monitors it.

YP4

However, a few young people and parents or carers preferred not to focus on pain if it is low-level:

I do not think that I would like that app as much because it is too pain orientated...implying that you are in constant pain...if you were just in minor pain and you were more interested in other things.

YP8

And I think that this might be a little bit too much adult...because she knows when she is in pain...I don’t know whether she would do that [use pain tracker].

PC1, parent or carer #1

In contrast, professionals mostly discussed young people’s condition monitoring in the context of information sharing to enable efficient use of clinic appointments. For example, an app was viewed as a better alternative to the current paper-based option for young people to report on the frequency or level of pain, and medication use, between appointments:

So, apps can certainly be used to gain and to assess information and bring it to clinic...also feedback in real time.

HCP2

Young people discussed information-sharing in the context of a mutual exchange of communication with professionals, suggesting for example:

Your doctor can write something in [the app] maybe that you can read. Or...automatically send some leaflets or something to your house.

YP4

Young people explored the usefulness of parents’ or carers’ access to the app. One young person suggested that, depending on their age, young people could have the option when entering data to decide whether to share it with their parents or carers:

So, the younger ones [children], their parents can look.

YP1

#### Facility for Reminders

All participants recommended a facility for sending reminders, for example, to take medication and attend hospital appointments. One young person suggested a medication reminder that "[c]ould remind you again a couple of days later" [YP5].

A parent or carer suggested:

You would have to get the appointment somehow, networked to the unit at the hospital for them to be able to put the information in.

PC3

Additionally, professionals recommended adding reminders to the app about issues they would normally put in a letter to the family:

You have a clinic appointment so please bring shorts, or dress appropriately...that I suppose will help us a lot.

HCP3

### Theme 2: Components and Content of Mobile Apps

#### Desired Components of the App

Professionals referred to existing apps that could be adapted for use by young people with Juvenile Arthritis. They discussed the usefulness of components such as mindfulness and relaxation techniques, as well as tools, to monitor pain and report treatment side effects. In addition, tools aimed at improving treatment adherence, such as schedules for regular investigations, were considered important for optimal management of Juvenile Arthritis. However, these were generally not seen as important by young people:

It is something that I would look at and then just...move it to one side.

YP1

Professionals, young people, and parents or carers unanimously recommended gamified apps. Professionals thought apps needed to be fun and interesting to help young people learn about their Juvenile Arthritis. Young people also favored quiz-styled learning activities and characters they could *adopt* as part of the app, such as a pet:

If you made it into a game like, if you had a character to help you learn it more and help you and stuff you could make it give you information and then you could feed and bath your pet and stuff. And, it answers your questions and it gives you like notifications every day to feed it and wash it and stuff and you can put your pain scales in and stuff like that. And, as well it takes your mind off of your pain as well.

YP7

Although one professional said that gamified apps can be suitable for all age groups, young people emphasized that it should be age-appropriate. Professionals also suggested that young people could receive digital currency or rewards for self-management activities, such as taking their medication:

Because there is no incentive to do apps unless you get a reward.

HCP3

Several young people favored competitive behavior through a gamified app, suggesting a leader board as an incentive to encourage others to use the app in self-management. However, professionals were concerned about introducing competition, as this could encourage young people to compare themselves with others, overlooking the unique nature of their conditions as:

You run the risk of them comparing themselves to someone else who is doing much better than they are.

HCP8

#### Essential Content

Information provision was viewed as an essential feature of an app specific for young people with Juvenile Arthritis. Information about the condition, treatments, symptom management, and how other young people are living with the condition were identified as relevant content. However, it was stated that information should be presented in an age-, gender-, and developmentally appropriate way, tailored for different reading abilities and learning styles and in the form of fun activities. For example, the professionals pointed out that:

It is not all about saving our time though and taking away things from what we are doing, it’s about engaging young people.

HCP4

All participants recommended that the style and format of presentation of materials be visually appealing to promote engagement with the app. Examples of ways to present information included *bite-size chunks* that could be built on video clips. The visual impact and interactivity was highly important to young people:

Something that is fun, engaging, interactional but educational. Fun but you learn something too...us teenagers, like we are quite picky and it’s the look...so if it’s just writing then it will be oh just, you know flick it through and get bored.

YP1

#### Practical Considerations of Apps

Several practical considerations were raised by respondents in relation to using the app. Safeguarding was an important issue for all participants, and it was suggested that the app was password protected, with professionals providing young people with the password. Requirements such as updating the app regularly and compatibility with new and existing mobile technology were expressed. For example, one parent or carer explained that:

I have had games in the past which will suddenly stop working and I know that it is the system because they have been updated past the point that, that system can cope with.

PC3

One young person suggested that the app contents should be backed up to a cloud storage facility so that the information could be downloaded to multiple devices with the appropriate permissions. Being able to access the app any time and across devices, with or without Wi-Fi or cellular data, was seen as important by young people and parents or carers, as not all young people have access to Wi-Fi.

All participants recognized the costs associated with developing an app, and although the availability of a free app was preferable, parents or carers and young people suggested that if there was a charge, a free trial was essential to enable them to self-assess whether the app was beneficial or not:

I think that you need to try before you buy. If you think...this is fantastic, it does not matter how much it is at that point, you will put your money into it because it works for you.

PC3

One young person suggested that existing reviews and comments from users would influence their decision to use an app. However, parents or carers’ views differed, with one individual suggesting that they would prefer to either pay upfront, or not at all. Professionals raised important issues about commissioning of the app within the current health care infrastructure. HCP2 referred to the need for discussion with commissioners regarding tariffs, particularly when considering the long-term use and applicability of such apps.

### Theme 3: App-Enabled Social Support (Emotional and Practical)

#### Access or Signposting to Existing Networks

All participants felt that an app should provide some form of social support. Professionals suggested signposting app users toward existing voluntary agencies offering information and support, such as:

JIA NRAS [Juvenile Idiopathic Arthritis, National Rheumatoid Arthritis Society] but it is for parents really, Arthritis Research UK and Arthritis Care.

HCP7

Several parents or carers and young people also thought signposting to social events would be important for young people as:

Then you can tell your parents about it and so maybe it could go through to your parents as well.

YP4

#### Secure Peer Support

Almost all young people suggested that the app should provide contact with others diagnosed with Juvenile Arthritis. For example, one young person explained that:

I didn’t really know anybody at all that had arthritis.

YP1

Young people consistently saw this as a way of sharing experiences as:

It would be really good to see what other people have gone through and if I can relate to them [YP6], ask: How do you guys cope? [YP1] and: Feel kind of welcome to this little group of ours.

YP5

Young people expressed a need for contact with others with the same condition, despite help given by parents or carers and professionals:

You know that they are really trying to help you but it makes you frustrated especially because they are not going through what you are.

YP4

Professionals and parents or carers also saw the value of peer support. For example, a professional pointed out that it “can be really helpful” [HPC8].

And one mother explained that her daughter:

Would know a lot more about what she has to face if she could talk to other children who have arthritis.

PC4

Professionals and parents or carers recommended a secure, moderated social network that young people could opt into with consent as “Many [with Juvenile Arthritis] feel isolated” [HCP3] and “Just being able to talk to someone is sometimes quite a big thing” [PC6]. All young people saw a chat room as a useful way to access peer support: “[a] group chat, that would be amazing” [YP1]. YP2 said, “If I am like getting worried about it (Juvenile Arthritis), I might go in and ask someone”.

Even a young person who firmly stated that they would not contribute to a chat room added, “But I could see like everybody else” [YP8].

However, it was recognized that: "A chat room would need to accommodate the different age groups” [PC4].

Issues relating to safeguarding emerged with young people raising security concerns, such as:

If somebody who did not actually have it [arthritis] but just pretended to have it to talk to children, well that could be a problem.

YP7

Repeated reference was made to the need for secure, moderated access as a way of restricting inappropriate users, although “Extra resources would be needed to maintain this” [PC5].

One parent or carer suggested the safer alternative of a message board or forum that enabled young people and parents to post and receive responses in real time and “could be easily monitored...by an administrator” [PC3].

#### Understanding From Others Without Juvenile Arthritis

Some means of educating peers about Juvenile Arthritis was requested by young people and parents or carers. For example, one young person explained that when taking tablets in the company of peers, everybody looked away so:

Something as depressing and serious as taking medication should be put in [the app]…it would be very useful for a lot of people including me.

YP6

Even professionals were seen to require more understanding as:

He [physio] said it is all up here, it is up in your brain...pain is an everyday thing.

YP1

Information provision in the form of an app was also viewed as a potential route for young people and their parents or carers to effectively explain the condition to other agencies, for instance the young person’s school:

You then could show them [teachers and peers] this app and that this kind of medication, sometimes it can do A, B and C to me.

YP5

#### Parent Support Network

That the app could also include some form of access and support for parents was referred to by parents or carers as they too could feel isolated, having only spoken to professionals:

I have never spoken to a mum or a dad [about Juvenile Arthritis, ever.

PC6

They explained that:

Some parents can find it more stressful to deal with than others.

PC4

So through such access:

You can share information and ideas with other parents as to how you have dealt with it.

PC4

Young people also recognized this need and suggested that:

You could have a little chat room between parents because it is hard work.

YP9

And it would be:

Something for parents in addition to young people so they can monitor and support their child.

YP1

## Discussion

### Principal Findings

Findings from this study suggest that the purpose of an app for self-management of Juvenile Arthritis should be to provide young people with the ownership and control of an interactive, engaging tool that gives information, monitors symptoms, offers reminders, and provides social support. Using the different elements of the app to manage their condition potentially enables young people with Juvenile Arthritis to develop a greater sense of autonomy through sharing the responsibility of managing their condition with professionals, as advocated by Foster et al [7], Schwartz et al [3], and Stinson et al [8]. Sharing access with professionals and discussing experiences recorded on the app can also help in developing good relationships between patients and professionals. Therefore, such an app will help young people with Juvenile Arthritis to develop the essential core skills of problem solving, decision making, resource utilization, and relationship formation with professionals, as identified by Lorig and Holman [2].

Cai et al [33] and Whitehead and Seaton [34] report similar findings to those emerging from this study and stress how such electronic devices have the potential to offer convenient ways of providing self-management interventions. Analysis of data from this third phase of our project also confirms the conclusions drawn from our previous survey [20] and supplements the findings of our previous systematic review [14]. Moreover, they meet the criteria currently established by Wyatt and Williams [35] that such an app should be evidence-based, up-to-date, interactive, age-specific, and include privacy precaution to promote self-management.

Young people’s request for an age-appropriate app with the potential for users to control the presentation also reflects the need for customization found within previous studies, such as that by Cai et al [33], Kenny et al [36], and Liptzin and Szefler [37]. The need for interactivity with potential rewards for good self-management to promote engagement identified by participants has also been reported by Cai et al [33]. This confirms the usefulness of games for self-management of chronic conditions [38-40], and the need to heed the advice of et al [41], who stress the importance of further work to identify potential gaming trends of the future. Such future work is important as the style of the game must be carefully designed because, although gamified apps are consistently downloaded by young people, inclusion of such facilities does not appear to influence the way many apps are rated in terms of popularity [41].

Some practical challenges were identified by participants including cost, compatibility with other technology, and the need to constantly update some of the content. Such concerns reflect those expressed in previous research, particularly given how the speed of technological advances may mean that modifications could be outdated even during development and evaluation and changes in equipment and functionality create difficulties when designing a mobile app [16].

That some form of access to peers should be included in an app has been found in previous studies, such as those by Cai et al [33] who recommend this should be accessed through signposting to existing organizations such as voluntary groups. Although such signposting was identified as useful in this study, there was considerable desire expressed by young people for the app to provide links with others diagnosed with Juvenile Arthritis through some form of chat room. Indeed, the number of comments offered by young people and parents or carers requesting peer-based social support was so noteworthy within this study that a second paper is planned to explore why this need is still so great, despite the support currently available from rheumatology clinics and voluntary groups. Only one young person did not see peer support as an important function of an app, but even this participant saw the value of observing how others were coping with their illness. Nevertheless, young people’s request for a chat room facility raised challenges related to safeguarding and monitoring contributions, also recognized by parents or carers and professionals, and requiring serious consideration when designing an app.

Two novel findings of this study are: the suggestion that an app could be used to help foster understanding from others without Juvenile Arthritis and accelerate the process of informing other agencies such as schools and social services and the request for a parent support network to be included in the app. Acknowledging input from parents could prove fruitful, particularly because they are still the most popular source of health information for young people [13].

### Limitations

Adopting a qualitative approach within this study has meant that it has some limitations. The limited opportunity within sampling to fully represent the number of young people in terms of age, gender, disease type, severity, longevity, and geographical location must be acknowledged. This was a single site study and for pragmatic reasons, it was only possible to invite young people diagnosed with vasculitis, uveitis, and JIA (systemic, polyarticular, and oligoarthritis) to participate. Moreover, it was only possible to invite 2 young males (10 to 12 years) from an ethnic minority background. One of these young males declined to take part, and the other did not complete the recruitment process. In addition, it was only possible to interview 2 of the 6 eligible male participants. Reasons given for not finally taking part in interviews included loss of interest and flare-up of illness.

Larger follow-up studies would enable inclusion of more males, ethnic minority groups, and other long-term conditions. This would be useful in case any cultural or gender differences could be identified which would supplement the findings reported here. Interestingly, Weiser [42] reported that young males use electronic media more for leisure and entertainment compared with young females who rather use it for educational purposes and interpersonal communication. Moreover, the participants of a study by Kenny et al [36] suggested that apps may provide an emotional outlet for males who may normally be reticent to display emotions.

However, the strength of this study is that it is part of a phased approach to development and evaluation of a complex intervention in line with the Medical Research Council framework [19]; it gathers the views of users, adopts a team approach, and includes user representation at all stages. Gathering data from interviewing young people and parents or carers together provided rich, detailed insight into their views about mobile apps and was not felt to inhibit contributions from younger participants. Indeed, it was noted that in each case, parents or carers consciously *took a back seat* and only offered information when asked, or prompted a young person when appropriate. It was particularly noteworthy just how the young people and their parents or carers enthusiastically encouraged each other in imagining different forms of informative games that could be included in the app.

Including young people and their parents or carers and professionals provided different perceptions of how mobile apps can aid self-management of Juvenile Arthritis. Although there was much agreement between the three groups, on some occasions, there was a difference in the degree of emphasis placed on some features of such an app. For example, professionals placed great emphasis on how shared information from an app could save time and staff engagement during consultations; however, young people and their parents or carers saw this facility more as allowing communication in the periods between consultations. Moreover, although peer support was a priority for young people, it was only mentioned briefly by professionals who may have already considered some of the concerns this may raise if included in the app.

Interestingly, although all professionals and parents or carers saw pain management as an essential feature, there were differences here between the views of young people. Some wanted pain monitoring, but others did not want this to be a dominant feature of the app as they did not want to be reminded of pain when not actually experiencing it. This has implications for the use of current pain apps by young people. It suggests that when including this feature in a self-management app, its usefulness be made clear, particularly regarding recording pain levels during the past and previous days and not just the current time. The opportunity to identify differences in the views between those treating and those living with Juvenile Arthritis that was enabled by this study, confirms the need for patient and HCP partnerships in all areas of care and health care research.

The completion of this modeling stage as a precursor to developing and evaluating an app paves the way for a future full trial that will include patient and professional representation within a team approach.

### Implications for Practice

Initially, introducing such an app could place more demands on health professionals treating those with Juvenile Arthritis. However, in the longer term, it is likely to alleviate their workload and improve communication and patients’ well-being and potentially influence a smoother transition process as young people will be better engaged in their health care. Rigorous, comprehensive commissioning for professional involvement and app moderation needs to be considered at the outset of app development.

### Conclusions

Agreement was found between young people with Juvenile Arthritis, their parents and carers, and professionals that a mobile app aiding self-management would be useful. This was provided that young people held a sense of ownership and control of an interactive, engaging device offering information and reminders as well as monitoring symptoms and providing social support. Findings justify and pave the way for a future feasibility study into the production and preliminary testing of such an app.

## Appendix

Multimedia Appendix 1 Topic Guides.

## Abbreviations

HCP: health care professional
LTC: long-term condition
NRAS: National Rheumatoid Arthritis Society
