# Supplementary material for: Young People’s, Parents’, and Professionals’ Views on Required Components of Mobile Apps to Support Self-Management of Juvenile Arthritis: Qualitative Study
Source: JMIR Mhealth Uhealth. 2018 Jan 19;6(1):e25. doi: 10.2196/mhealth.9179 (PMC5797289; doi:10.2196/mhealth.9179)
Supplement: Multimedia Appendix 1 [file mhealth_v6i1e25_app1.pdf]

Self-Management Mobile App for young people with Juvenile Arthritis

Information base underpinning the rationale for running focus groups:

- Mobile phones and tablets can function with custom software programs called apps, which technologically allow the development of condition-specific and patient-tailored software. These are personal devices, adapted by the user (to a certain degree) to reflect their specific needs, thus allowing for adaptive, responsive, confidential, and targeted channels of communication and alerts.

- Five core skills in the personal management of chronic physical conditions: Problem solving, Decision making, Resource utilization, Develop patient-health professional relationships, and Taking action.

The aims of focus groups:

- To explore how apps can support these skills through by providing and collecting information in an accessible, convenient, and interactive way.

- To investigate the potential embedding gamification into the app, taking elements of game design such as badges and levels, to encourage engagement with the information and particular behaviours.

- To explore how apps can facilitate communication between patients and professionals, social networking, capturing real-time data, monitoring bodily functions, automated feedback, guidance and clinical alerts, and smart decision-making tools.

- To gain awareness of how much mobile phones and tablets are used by participants.

Topic Guide: Healthcare Professional Group

The aim of this group will be to explore healthcare professionals' views about what should be included in an app for mobile phones which would help in managing their chronic rheumatic disease. Other focus groups will include young people aged between 10 and 18 years and parents of young people with CRD.

Views will be sought on the advantages and disadvantages of existing mobile apps as forms of information and support regarding both specific and generic chronic disease issues. Exploration will also be made of where healthcare professionals think that young people look for information about their health, the reasons why they use that information and feasibility of gamification (using game based techniques) for compliance.

The following topic guide is designed to stimulate debate, gain insight and generate ideas to pursue in greater depth and includes demonstrations of existing mobile apps. Topic guides will be regularly refined to reflect emerging issues.

Welcome and context-setting - the facilitator will introduce themselves explaining who they are and what their role is.

- They will introduce the overall aim of the study.
- They will introduce the objectives for the focus group session (as mentioned above).
- They will explain that an audio voice recorder is being used to record the discussion but participants will not be identifiable to anyone other than the researcher and the Principle Investigator.

The facilitator will then explain the 'ground rules' for the session:

- Confidentiality is important and whatever is said in the room stays in the room and is not repeated outside to other people.
- There are no right or wrong answers.
- Everyone will have a chance to speak and be heard.

The facilitator will explain how information will be used/shared.

- How findings will be fed back to the research sponsor and participants on the project.
- The facilitator will check that everyone is happy to continue and ask if there are any questions from the group.

Health information needs – general discussion

The facilitator will ask the group members:

- To describe any information needs their patients have in relation to managing chronic rheumatic disease and how these should be presented.
- To describe what information sources their patients already use,
- If these sources are effective
- How they may be improved, particularly if offered in a gaming format (gamification).

Opinions on current mobile apps available

The facilitator will split the group into smaller sub-sections.

- Healthcare professionals in each sub-section will be asked to examine the examples of existing self-management mobile apps.
- Professionals from each subsection will then be asked by the facilitator to report back to the whole group their likes, dislikes and suggestions regarding each app.
- This will help to facilitate further discussions and inform further research into designing a mobile self-help app.

Suggestions for what self-management apps should include

The facilitator will ask the group:

- For examples of what kind of information they think young people such as their child need in a mobile app to help in self-management of chronic rheumatic disease.
- How this information should be presented
- What tools would be useful for self-management, such as recording pain and the weather, and interactive VAS scale for physical and emotional feelings.

Feedback

The facilitator will:

- Encourage comment/response from the different group members regarding the information presented, mentioning what challenges *and opportunities exist.*
- Thank the group for their attendance and assure them that their contribution will help to inform the feasibility of developing a mobile app.
- Ask them if it would be acceptable to contact them regarding a follow on phase to test out such an app.

The facilitator will ask the group how they found the process.

Topic guides for interviews

A similar procedure is planned to that of focus groups to accommodate participants who prefer individual interviews. The wording will be changed to be appropriate, depending on if it was a young person, parent/carer or healthcare professional but the procedure will follow the same format as that for focus groups:

Welcome and context-setting

The researcher will introduce themselves explaining who they are and what their role is in the study**.** They will introduce the overall aim of the study and the aims of the interview:

- To explore how apps can support these skills through by providing and collecting information in an accessible, convenient, and interactive way. To investigate the potential embedding gamification into the app, taking elements of game design such as badges and levels, to encourage engagement with the information and particular behaviours.

- To explore how apps can facilitate communication between patients and professionals, social networking, capturing real-time data, monitoring bodily functions, automated feedback, guidance and clinical alerts, and smart decision-making tools.

- To gain awareness of how much mobile phones and tablets are used by participants

- They will explain that an audio voice recorder is being used to record the interview only accessible to the researcher and the Principle Investigator.

The researcher will explain how information will be used/shared.

- How findings will be fed back to the research sponsor and participants on the project.

- The facilitator will check that the participant is happy to continue and wishes to ask any questions.

Health information needs – general discussion

The researcher will ask the participant:

- To describe any information needs in relation to managing chronic rheumatic disease and how these should be presented.

- To describe what information sources already used

- If these sources are effective

- How they may be improved, particularly if offered in a gaming format (gamification).

Opinions on current mobile apps available.

- The participant will be asked to examine the examples of existing self-management mobile apps.
- They will then be asked about their likes, dislikes and suggestions regarding each app.

Suggestions for what self-management apps should include.

The researcher will ask the participant:

- For examples of what kind of information they think young people need in a mobile app to help in self-management of chronic rheumatic disease.
- How this information should be presented
- What tools would be useful for self-management, such as recording pain and the weather, and interactive VAS scale for physical and emotional feelings.

Feedback

The researcher will ask the participant for:

- Comments about what challenges and opportunities exist.
- Thank them for their attendance and assure them that their contribution will help to inform the feasibility of developing a mobile app.
- Ask them if it would be acceptable to contact them regarding a follow on phase to test out such an app.

****
